# Changes in Soil Microbial Community Structure and Assembly Process Under Different Forest Restoration Strategies in Cold Temperate Forests of Northeastern China

**DOI:** 10.3390/microorganisms13061339

**Published:** 2025-06-09

**Authors:** Rongze Luo, Mingyu Wang, Youjia Zhang, Hong Wang, Xiangyu Meng, Xin Gao, Yuhe Zhang, Xin Sui, Maihe Li

**Affiliations:** 1 Engineering Research Center of Agricultural Microbiology Technology, Ministry of Education & Heilongjiang Provincial Key Laboratory of Ecological Restoration and Resource Utilization for Cold Region & Key Laboratory of Microbiology, College of Heilongjiang Province & School of Life Sciences, Heilongjiang University, Harbin 150080, China; hellolrz@163.com (R.L.); wmy022234@163.com (M.W.); zdzhidian@outlook.com (Y.Z.); 13163400129@163.com (H.W.); m13555220601@163.com (X.M.); agaoxin0218@163.com (X.G.); 13703670569@163.com (Y.Z.); 2 Forest Dynamics, Swiss Federal Institute for Forest, Snow and Landscape Research, CH-8903 Birmensdorf, Switzerland; 3 Key Laboratory of Geographical Processes and Ecological Security in Changbai Mountains, Ministry of Education, School of Geographical Sciences, Northeast Normal University, Changchun 130024, China; 4 School of Life Science, Hebei University, Baoding 071002, China

**Keywords:** microbial community diversity, nature restoration, artificial restoration, community structure and assembly

## Abstract

The cold temperate forest ecosystem is a crucial ecological zone in China, significantly impacted by human activities. To understand the impact of restoration on soil microbial communities following disturbance, this study employed high-throughput sequencing technology to systematically examine the assembly patterns and processes of soil microbial communities under two restoration modes (nature restoration (NR) and artificial restoration (AR)) in this forest ecosystem. The results indicated that the concentrations of total nitrogen (TN), alkaline hydrolysable nitrogen (AN), dissolved organic carbon (DOC) and soil organic carbon (SOC) were significantly higher in soils under natural restoration compared to artificial restoration. The α-diversity of soil bacteria remained unchanged, while soil fungal α-diversity changed significantly across different restoration modes. Furthermore, different restoration modes significantly alter the β-diversity of soil microbial (bacterial and fungal) communities. The relative abundance of soil microbial (bacterial and fungal) changed significantly across different forest restoration strategies, i.e., the relative abundance of *Pajaroellobacter* increased in natural restoration compared to that in natural forest; similarly, both *Podila* and *Russula* showed higher relative abundances in natural restoration than those in natural forest. Furthermore, analysis of variance for differences between groups shows that *Incoybe* plays a crucial role in artificial restoration. Community assembly analyses indicated that that soil microbial (bacterial and fungal) communities were primarily driven by deterministic processes in both restoration models. In short, our study improves our comprehension of how soil microbial communities respond to different restoration methods in temperate forest ecosystems, providing valuable insights for their sustainable management.

## 1. Introduction

The cold temperate forest ecosystem is one of the most important terrestrial ecosystems, playing a major role in biogeochemical cycles, climate regulation and ecological services [1]. Human activities, such as logging and forest fires, often disrupt forest ecosystems, leading to issues like reduced biodiversity, weakened climate regulation and increased soil erosion [2]. Due to the demands of economic development, the Daxingan Mountains experienced large-scale deforestation until 1998, resulting in a drastic reduction in the area of primary forest [3]. Since then, the Chinese government has implemented important forest policy reforms, including the complete cessation of commercial logging, and has assessed the long-term environmental impacts of these policies, particularly in the areas of forest restoration, biodiversity conservation and timber production [4]. It is worth noting that conservation mainly involves the artificial cultivation of pioneer tree species, such as birch forests, and partly relies on natural restoration [5,6]. Therefore, understanding the structure and function of artificial and naturally restored forest ecosystems is important for managing the forests.

Natural restoration refers to the restoration of degraded ecosystems with no or as little human intervention as possible, relying primarily on the forces of natural succession [7]. Artificial restoration refers to the restoration of natural ecosystems using artificial means to simulate the organizational structure of natural ecosystems based on ecological principles [8]. Due to the different contributions of biodiversity and ecosystem services of different forest restoration methods, previous studies have compared the effects of natural and planted forest restoration. For example, Hua et al. found that natural forests were significantly better than planted forests in terms of biodiversity, carbon storage, soil conservation and water retention, while planted forests had advantages only in timber production [9]. Additionally, Gao et al. found that natural restoration can effectively increase the nitrogen and organic carbon content of soils compared to artificial restoration [10]. However, there is no consensus on the effects of natural and artificial restoration on soil microorganisms, and changes in soil microbial communities respond differently in different forest types.

Soil microbes, as important indicators of forest ecosystem health, play a regulatory role in ecosystem energy flow and material cycling. Through their complex structural composition and dynamic assembly mechanisms, they maintain the stability of ecosystem services and functions under environmental change [11,12]. Controlling the soil microbial communities that assemble is essential to elucidate both the microbial succession of different restoration processes and the ecological processes of aboveground plant succession [13]. Current research on forest soil microorganisms focuses on the effects of land use change on soil microorganisms, the effects of nitrogen deposition on soil microbial structure, and the interaction between plant diversity and soil microbial diversity [14]. Despite the importance of soil microbial communities in forest ecosystems, little research has been conducted on their assembly mechanisms under different restoration regimes. Therefore, investigating how forest soil microbial communities assemble in different restoration contexts can provide critical insights into the complexity of forest ecosystems.

The Shuanghe National Nature Reserve in Heilongjiang Province aims primarily to conserve typical boreal forest ecosystems. The reserve contains dense old-growth forests and extensive wetlands, which are characterized by a variety of forest types and species richness, contributing to high biodiversity. In addition, the area includes extensive stands of naturally and artificially restored *Betula platyphylla* Suk. forests, offering valuable insights into the assembly of soil microbial communities under different restoration regimes. We hypothesize that: (1) the α-diversity and composition of soil microbial communities differ between different forest ecosystem restoration modes; (2) the mechanisms of soil microbial community assembly change with different restoration processes. Our aim is to improve our understanding of natural and engineered restoration, especially in relation to long-term recovery. This study is crucial for ecological research and has important implications for sustainable land management in forest ecosystems.

## 2. Materials and Methods

### 2.1. Research Area

This study was conducted in the Shuanghe Nature Reserve (52°56′27″–53°11′04″ N, 124°57′56″–125°15′10″ E) in Heilongjiang Province. The region has a cold-temperate continental monsoon climate, with an average annual temperature of −4.3 degrees Celsius, an extreme low of −45.8 degrees Celsius and an extreme high of 38 degrees Celsius. Winters are cold and long, with an average of 170 snow days per year and an average snow depth of 20–50 cm. Summers are rainy, especially in July and August, and average rainfall is 70–150 mm, experiencing roughly 460 mm of annual precipitation. The lowest temperatures are in January and the highest in July. The Reserve has dense primary forests, extensive wetlands and a rich variety of wildlife, contributing to a high level of biodiversity. The vegetation is characterized by cold temperate species, with diverse and abundant forest types, particularly large areas of original *Betula platyphylla* Suk. and *Larix gmelinii* forests.

### 2.2. Experimental Design and Sample Collection

To investigate soil microbial composition, diversity and community assembly patterns under different restoration regimes, we set up three independent plots (10 m × 10 m) in each restoration vegetation type (naturally mature (CK) *B. platyphylla* forest, a naturally restored (NR) *B. platyphylla* forest and an artificially restored (AR) *B. platyphylla* forest), and the distance between any two plots was >50 m on 10 September 2024. A five-point composite sampling technique was implemented to procure soil specimens, consisting of three aseptic soil cores retrieved from the 0–20 cm organic stratum, each measuring 5 cm in diameter and extending to a 20 cm depth. Subsequent to the excision of superficial detritus and humic strata, approximately 1 kg of soil material was harvested from each designated sampling plot. The collected soil underwent mechanical sieving through a 2 mm aperture mesh to eradicate gross contaminants such as mineral fragments, discernible rhizomorphic structures, and organic residues. Following purification, each sample was bifurcated into dual subsamples: the first portion was preserved under cryogenic conditions at −80 °C in preparation for microbial community sequencing, whereas the second aliquot was maintained at a refrigerated temperature of 4 °C to facilitate comprehensive assessment of soil physiochemical characteristics including moisture content, pH, organic carbon concentration, and nutrient profiles. This methodological approach ensures both representative sampling across spatial gradients and appropriate preservation protocols to maintain soil integrity for subsequent multi-dimensional analyses.

### 2.3. Determination of Soil Chemical Properties

Soil pH measurements were conducted with a calibrated pH meter (Thermo Scientific Orion, Cambridge, UK) [15]. To prepare the samples, large particulate matter and debris were removed via mechanical sieving using a 2 mm mesh. Before each use, the pH meter was calibrated with standard buffer solutions (pH 4.01, 7.00, and 10.01) following the manufacturer’s instructions. The soil sample was combined with distilled water in a 1:2.5 (*w*/*v*) soil-to-water ratio, stirred thoroughly and left to equilibrate for 30 min. The calibrated instrument was used to measure the pH of the supernatant, with duplicate readings obtained to guarantee the precision of the data. TN and SOC concentrations were quantified using an elemental analyzer (Elementar Vario EL III, Elementar, Germany) [16]. This instrument employs high-temperature combustion technology, eliminating the need for hazardous reagents such as concentrated sulfuric acid, catalysts or hydrogen peroxide, which are typically required in traditional Kjeldahl nitrogen analysis. The results were recorded upon completion of the analysis and used to calculate the TN and SOC content of each sample with high accuracy. This method ensures reliable quantification of key soil parameters while minimizing chemical waste and procedural complexity. Soil organic carbon is oxidized to carbon dioxide through a chemical oxidation process involving a potassium dichromate-sulfuric acid solution. The soil DOC content was then measured using either titration or turbidimetry. This approach relies on the selective oxidation of organic matter by the chemical reagent, with the extent of oxidation quantified via titration against a standard solution or by measuring the turbidity of the reaction mixture [17]. Ammonia in the absorbent was titrated with a standard acid solution (hydrochloric acid solution) and the AN content of the soil was calculated from the volume of acid consumed in the titration. Effective available phosphorus (AP) was assessed through colorimetry following extraction with 0.5 M NaHCO_3_ solution [18]. Quick-acting potassium (AK) content was extracted using acetic acid and ammonium leaching [19]. Three independent replications were carried out for each sample for all soil chemical properties.

### 2.4. DNA Extraction

Extraction of total soil DNA was performed on 0.5 g of soil, following the protocol specified in the E.Z.N.A. Soil DNA Kit (Omega Bio-tek, Norcross, GA, USA) [20]. The amplification of the V3-V4 region of the bacterial 16S rRNA gene was conducted using the forward primer 338F (5′-actcctacgggaggcagag-3′) and the reverse primer 806R (5′-ggactachvgggtwtctaat-3′). For the fungal ITS rRNA gene, the ITS1 region was targeted with the primer pair ITS1 (5′-cttggtcatttagaggataa-3′) and ITS2 (5′-gctgcgttcatcgatgc-3′) [21]. DNA quantity and quality were assessed via spectrophotometry. The 10 μL PCR reaction mixture contained 0.5 μL template, 5 μL KOD FX Neo buffer, 2 μL dNTPs (2 mmol/L each), 0.3 μL of each primer (10 mmol/L), 0.2 μL KOD FX Neo, and double-distilled water to 10 μL [22]. The bacterial PCR amplification was performed under the following conditions: an initial denaturation step at 95 °C for 5 min, followed by 25 cycles consisting of denaturation at 95 °C for 30 s, annealing at 50 °C for 30 s, and elongation at 72 °C for 40 s. A final extension step was conducted at 72 °C for 7 min to ensure complete synthesis of the amplified products. PCR amplification of fungal DNA included a pre-denaturation phase at 95 °C for 5 min, followed by 25 sets of cycles. Each cycle consisted of denaturation at 95 °C for 30 s, annealing at 50 °C for 30 s and extension at 72 °C for 30 s. To ensure reproducibility, three replicates of each PCR reaction were carried out and the products were then combined. The amplified products were then subjected to electrophoresis on a 1.5% agarose gel to confirm the presence of the target band and to assess the quality of the PCR products. Subsequently, the PCR products were purified using the AxyPrep DNA Gel Extraction Kit (AxyPrep Biosciences, Union City, CA, USA). The purified DNA was quantified with a Qubit 3.0 fluorometer (Life Invitrogen, Waltham, MA, USA) to ensure accurate quantification. The samples were then normalized based on the required sequencing depth and combined in appropriate proportions for downstream sequencing analysis [23]. High-throughput sequencing was performed using the Illumina MiSeq platform (Shanghai Mingzhi Biomedicine Technology Co., Ltd., Shanghai, China). Taxonomic characteristics of bacteria were determined using the Silva v132 database (http://www.arb-silva.de) (accessed on 2 December 2024), which provides comprehensive and quality-checked ribosomal RNA sequence data for microbial classification. For fungal classification, the Global Fungi database (https://globalfungi.com/) (accessed on 2 December 2024) was employed, offering a specialized resource for identifying fungal taxa.

### 2.5. Bioinformatics Analysis

Raw fastq reads for bacteria and fungi were filtered and analyzed using QIIME1 (version 2022.2) [24]. The PEAR software (Pair-End ReAd MergeR, version 0.9.6) was utilized to merge forward and reverse reads. In order to improve the accuracy of the analysis, sequences were excluded if they were less than 200 bp in length or had an average quality score under 20 [25]. The trimmed sequences underwent chimera detection using the Uchime algorithm, and sequences identified as containing chimeras were subsequently removed. Additionally, sequences with overlapping regions exceeding 10 base pairs were excluded to maintain sequence integrity. The cleaned sequences were then subjected to clustering using the UPARSE algorithm, which groups sequences sharing ≥97% similarity into Operational Taxonomic Units (OTUs). This clustering threshold of 97% similarity is conventionally associated with species-level classification, providing a standardized approach to categorize microbial diversity [26]. The clustering process began with pre-processing steps that involved deduplicating and quality-controlling all sequence reads. These reads were then classified into distinct OTUs according to the defined similarity threshold. The UPARSE was employed to extract OTU sequences from the high-throughput sequencing data. In the analysis, the SILVA database (v.138) served as a reference for assigning specific taxonomy to bacterial and fungal OTUs. Additionally, the FUNGuild database was utilized to determine fungal functional guild assignments, providing insights into the ecological roles of fungal communities [27]. The total data set for all samples was subsampled to achieve a uniform sequence count, based on the lowest count observed, to ensure comparability for community analysis.

### 2.6. Statistical Analysis

The “vegan” package in R software (v.4.4.1) was utilized to carry out ANOVA analysis, assessing differences in soil physicochemical properties and the α-diversity of soil bacteria and fungi [28]. Furthermore, The α-diversity of soil microorganisms in each individual sample was determined by leveraging the ‘vegan’ package available in R software (v.4.4.1) [29]. Principal Coordinates Analysis (PCoA) was conducted using the “vegan” package in R, based on Bray–Curtis dissimilarities calculated at the OTU level [30]. Mantel tests were conducted using the ‘vegan’ package in R to assess the relationships between soil microbial community composition, soil physicochemical properties and soil microbial α-diversity [31]. Canonical Correspondence Analysis (CCA) was conducted using the microeco package in R software (v.4.4.1), based on the OTU table and soil physicochemical parameters. To further clarify the microbial community assembly processes and explore the fundamental mechanisms behind soil microbial community assembly, the Sloan neutral community model was utilized. This model is available for acquisition from CRAN at https://cran.r-project.org/ (accessed on 10 January 2025) [32]. LefSe plots were generated using the “ggplot2” package and abundance radar plots in R software (v.4.4.1) [33]. Circos plots were generated using the ‘circlize’ package in R software (v.4.4.1) [34]. Venn diagrams were generated using the “ggvenn” package in R software (v.4.4.1) [35]. Evolutionary trees (heat trees) for species classification were plotted using the “metacoder” package in R software (v.4.4.1) [36]. Correlation heat maps were visualized using the “corrmorant”, “pheatmap”, “ggplot2”, “ggcorrplot” and “corrgram” in R software (v.4.4.1) [37]. The analysis of significance maps between groups was performed by utilizing SPSS software (v.26), with the Kruskal–Wallis rank sum test serving as the basis for plotting [38].

## 3. Results

### 3.1. Soil Chemical Properties

The soil chemical properties of naturally restored (NR) and artificial restoration (AR) forests both differed significantly compared to those of naturally mature forests (CK) (Table 1; *p* < 0.05). The concentrations of SOC, DOC, TN and AN were highest in the CK and lowest in the AR. However, the concentrations of AP and AK were highest in the AR and lowest in the NR (Figure S6). The pH value of NR was highest, while that of AR was lowest (Figure S7). Overall, these differences indicate that different restoration methods have different effects on forest soil chemistry.

### 3.2. Soil Microbial Diversity and Composition

The α-diversity indices of soil bacterial communities did not change among the three different treatments (Table 2, *p* < 0.05), while the Simpson and Shannon indices of the soil fungal communities in the NR were significantly higher than those in the CK and AR (Table 2, *p* < 0.05).

**Figure 1:**
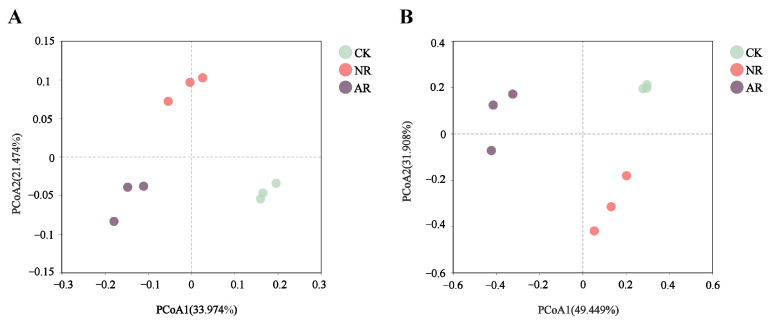
The figure presents the results of principal coordinates analysis (PCoA) for ecological sample similarity based on the Bray–Curtis distance calculations, showing bacteria (**A**) and fungi (**B**). The distance between samples, which denotes their similarity, is represented in a multidimensional space, with each point corresponding to a sample. Through PCoA analysis, high-dimensional data is projected onto a two-dimensional plane for easier visualization and understanding. Different colors of points are used to distinguish between sample groups, emphasizing the relationships both within individual groups and between different groups. CK: Naturally mature forests. NR: Naturally restored forests. AR: Artificially restored forests.

Principal coordinate analysis (PCoA), based on the Bray–Curtis distance, clearly showed that different restoration treatments significantly affected the β-diversity of soil bacterial and fungal communities (Figure 1). For soil bacteria, the first (PCoA1) and second (PCoA2) principal coordinates accounted for 33.974% and 21.474% of the total variance, respectively (Figure 1A). For soil fungi, PCoA1 accounted for 49.449% and PCoA2 for 31.908% of the total variance (Figure 1B). In addition, different restoration modes led to the disappearance or appearance of certain soil microbial genera (Figure S3).

**Figure 2:**
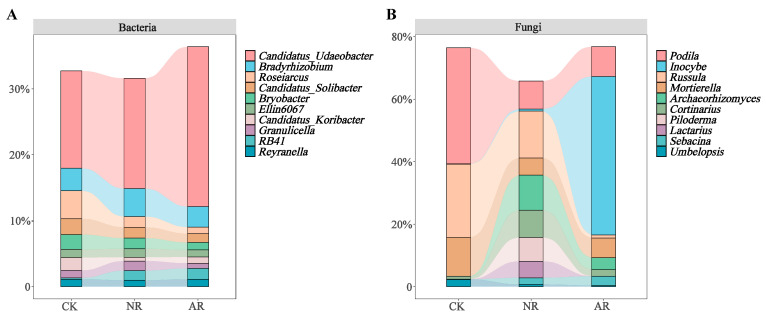
The top ten abundant soil bacterial genera (**A**) and soil fungal genera (**B**) under varying treatments are illustrated by stacked bar charts. CK: Naturally mature forests. NR: Naturally restored forests. AR: Artificially restored forests.

**Figure 3:**
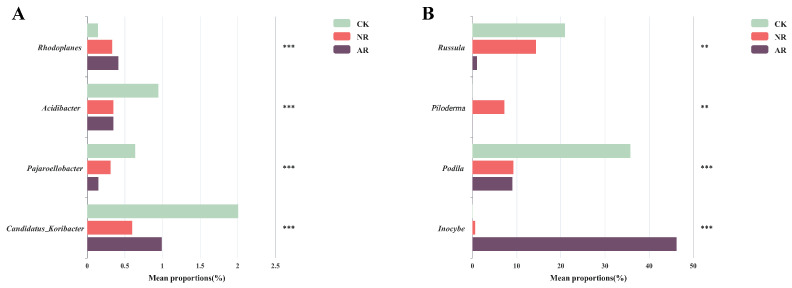
The significant difference test chart between groups was used to test the differences of bacteria (**A**) and fungi (**B**) under different recovery modes. Bar graphs with different colors represent different treatment groups (‘**’ indicates 0.001 < *p* ≤ 0.01; ‘***’ indicates *p* ≤ 0.001). The ordinate depicts different genus names, while the abscissa indicates the percentage of genera across various treatment groups. NR: Naturally restored forests. AR: Artificially restored forests.

**Figure 4:**
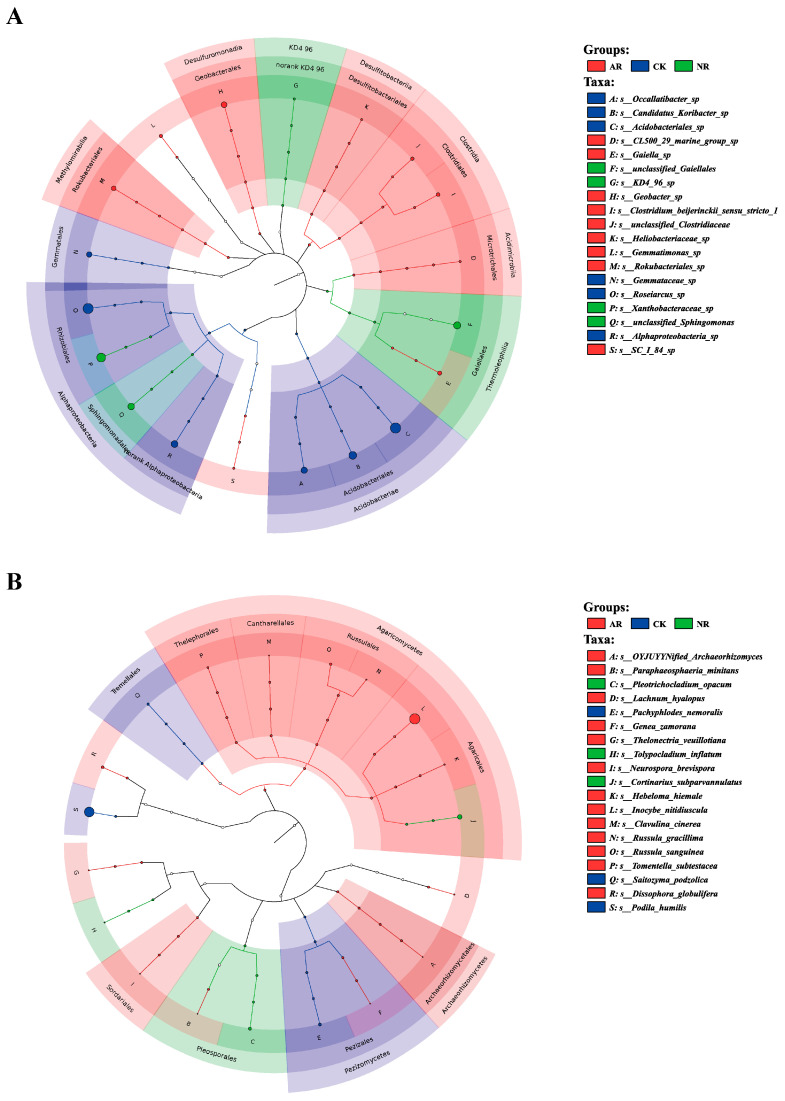
The LEfSe (Linear discriminant analysis effect size) results are visualized utilizing a circular tree diagram for soil bacteria (**A**) and fungi (**B**). Each color in the diagram corresponds to a distinct treatment group. Nodes in different colors along the branches represent microbial taxa that are significantly associated with the corresponding group, whereas yellow nodes indicate taxa without significant associations. The diagram’s right-side legend explains the English letters denoting species names. Consistent colors throughout the tree suggest the absence of significant markers between groups. CK: Naturally mature forests. NR: Naturally restored forests. AR: Artificially restored forests.

Different restoration modes significantly change the relative abundance of soil bacterial and fungal genera, such as *Candidatus_Udaeobacter* and *Roseiarcus* among the soil bacterial genera, and *Podila* and *Russula* among fungi, which differed significantly among restoration modes (Figure 2 and Figure S4). Notably, the relative abundance of *Rhodoplanes* increased significantly with the transition from CK to NR and AR, whereas the relative abundance of *Pajaroellobacter* decreased significantly (Figure 3A). The relative abundance of *Podila* and *Russula* decreased significantly along with CK to NR and AR (Figure 3B). Furthermore, linear discriminant analysis effect size (Lefse) analysis revealed that there were significant differences in the relative abundances of 40 bacterial taxa and 32 fungal taxa between the three groups (Figure 4). As can be seen from the histogram of the phylum levels of bacteria, Verrucomicrobiota had a higher relative abundance in AR. Proteobacteria had higher relative abundance in all treatment groups, but slightly lower in AR (Figure S5A). For the fungal community, Basidiomycota was the most dominant fungal phylum in all treatment groups, with the highest percentage particularly in AR. Mortierellomycota had a significant relative abundance in all treatment groups, but was slightly higher in NR (Figure S5B). A variety of bacterial functional genes are listed in Figure S8, including genes related to primary metabolism, secondary metabolism, signal transduction, transporter systems, and environmental adaptation. A variety of fungal ecological functional types, including plant pathogens, animal pathogens, saprophytes, and mycorrhizal fungi, are listed in Figure S9. The heat map demonstrated the similarities and differences in the abundance of microbial functional types among different treatment groups via cluster analysis, revealing the effect of forest restoration strategies on the functional diversity of soil microbial communities. These results demonstrate that the structure and composition of soil microbial communities vary depending on the restoration method employed.

### 3.3. Drivers Affecting Soil Microbial Communities

**Figure 5:**
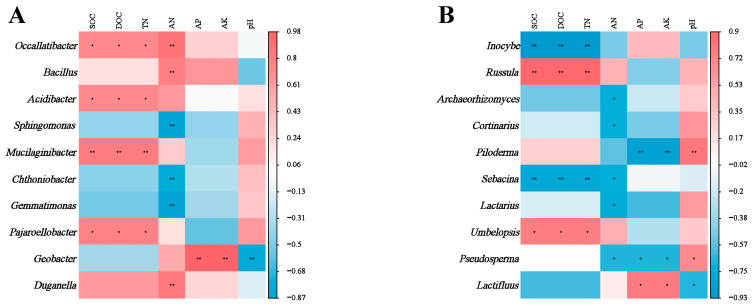
Significance levels (* for 0.05 and ** for 0.01) are indicated in the analysis of correlations between soil bacterial (**A**) and fungal (**B**) genera and environmental factors across various plant types. A color gradient from red to blue shows the strength of these correlations, with red meaning a positive correlation and blue meaning a negative correlation.

There was a strong correlation between soil microbial genera and soil chemical properties. The relative abundance of bacterial genera *Occallatibacter*, *Mucilaginibacter* and *Pajaroellobacter* correlated positively with SOC, DOC and TN (Figure 5A). Soil AN correlated significantly with most bacterial genera. In contrast, the relative abundance of *Geobacter* correlated positively with soil AP and AK, but negatively with pH. At the fungal genus level (Figure 5B), the relative abundance of *Incoybe* and *Sebacina* correlated negatively with SOC, DOC and TN. Conversely, the relative abundance of *Russula* and *Umbelopsis* correlated positively with SOC, DOC and TN.

**Figure 6:**
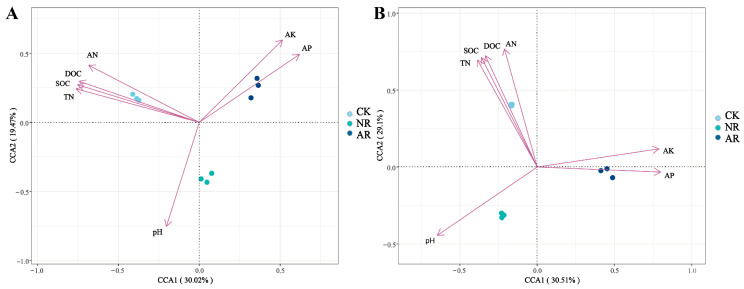
Canonical correspondence analysis (CCA) was utilized to explore the relationship between soil bacterial (**A**) and fungal (**B**) community structures, as represented by distinct OTUs, and key environmental variables including TOC, TN, AN, AP and pH. In the two-dimensional CCA plot, the spatial distribution of sample points, which are differentiated by shapes or colors to denote various treatment groups or sampling points, reflects the similarity in microbial community composition. The vectors (arrows) in the plot represent environmental factors, with their length indicating the magnitude of influence on community structure and their direction indicating the axis of maximum variation. The angle between two arrows signifies the correlation between the corresponding environmental factors: an acute angle denotes a positive correlation, whereas an obtuse angle indicates a negative correlation. CK: Naturally mature forests. NR: Naturally restored forests. AR: Artificially restored forests.

**Figure 7:**
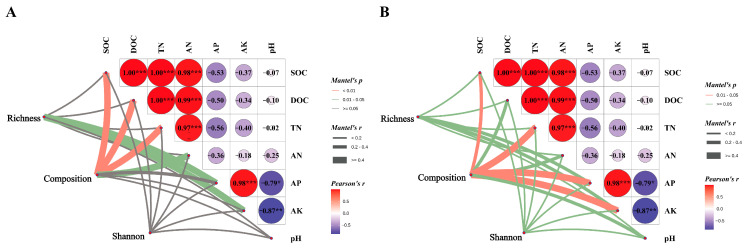
Through Mantel analysis, the relationships between soil bacterial (**A**) and fungal (**B**) richness, community composition, Shannon index, and soil physicochemical properties were elucidated. The red and green lines indicate varying degrees of correlation (‘*’ indicates 0.01 < *p* ≤ 0.05; ‘**’ indicates 0.001 < *p* ≤ 0.01; ‘***’ indicates *p* ≤ 0.001), while gray lines denote the absence of correlation. The thickness of the lines (i.e., the Spearman correlation coefficient) represents the magnitude of the correlation; thicker lines indicate stronger correlations, whereas thinner lines indicate weaker ones. Among these variables, TOC stands for total organic carbon in soil, TN for total nitrogen, AN for available nitrogen (alkali-hydrolysable nitrogen), AP for available phosphorus, and pH for the soil’s acidity or alkalinity. Richness refers to soil microbial richness, composition to the Bray–Curtis dissimilarity among microorganisms, and Shannon to the Shannon index.

Canonical correspondence analysis (CCA) was employed to elucidate the relationships between soil microbial community structure and environmental variables within the soil. The findings indicated that SOC, DOC, TN and AN exhibited positive associations with the soil bacterial community structure in the CK treatment, whereas they showed negative associations with the soil bacterial communities in the NR and AR treatments (Figure 6A). A comparable pattern of correlation was observed between soil fungal community structure and soil environmental factors, as depicted in Figure 7B. The Mantel test results further corroborated the existence of significant correlations between soil microbial community composition and key soil physicochemical properties, including SOC, DOC and TN (Figure 7). Additionally, bacterial richness was found to be associated with soil AP and AK. These results collectively underscore the influence of soil properties on shaping microbial community structures and highlight the distinct responses of bacterial and fungal communities to varying environmental conditions. These results demonstrate the complex interplay between soil microbial communities and soil chemical properties.

### 3.4. The Community Assembly Process of Soil Microbial Communities

**Figure 8:**
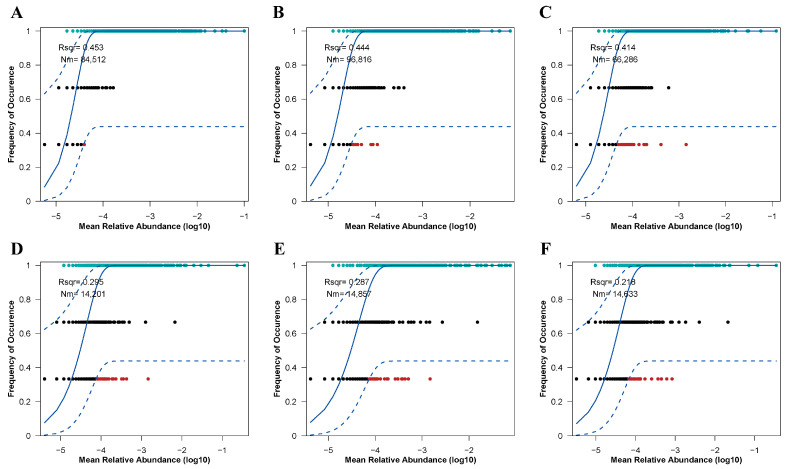
The neutral community model (NCM) was employed to fit soil bacterial ((**A**): CK, (**B**): NR, (**C**): AR) and fungal ((**D**): CK, (**E**): NR, (**F**): AR) communities under treatments with added organic nitrogen (urea). R^2^ represents the goodness-of-fit of the model. Nm denotes the product of metacommunity size (N) and migration rate (m) (Nm = N × m), which quantitatively estimates the degree of dispersion among communities and determines the correlation between occurrence frequency and regional relative abundance. The blue solid line represents the best fit to the neutral community model (NCM), while the blue dashed lines indicate the 95% confidence interval predicted by the NCM. Operational taxonomic units (OTUs, represented as black dots) within the confidence interval are considered to follow a neutral distribution.

The assembly processes of soil microbial (bacterial and fungal) communities under varying restoration modes were assessed utilizing the Sloan neutral community model (Figure 8). These restoration approaches significantly influenced the assembly mechanisms of both bacterial and fungal communities, primarily by altering the balance between deterministic and stochastic processes. For soil bacterial communities (Figure 8A), deterministic processes played a dominant role in community assembly. However, in the CK treatment, since R^2^ is less than 0.5, bacterial community assembly exhibited a tendency towards stochastic processes. Notably, the NR treatment demonstrated the strongest dispersal capacity among the bacterial communities (Nm = 96,816). Interestingly, soil fungal communities followed a similar pattern (Figure 8B), with their assembly processes being even more strongly governed by deterministic factors compared to bacterial communities (R^2^ < 0.3). The NR treatment also exhibited the highest dispersal efficiency within the fungal communities. These findings highlight the differential impacts of restoration modes on microbial community assembly and underscore the varying roles of deterministic and stochastic processes in shaping bacterial and fungal communities under different ecological conditions. The results provide insights into how restoration practices can influence microbial community structure and function in soil ecosystems.

## 4. Discussion

### 4.1. Effects on Microbial Diversity (α and β)

In the context of the different restoration modes considered in this study, the physicochemical properties of the soil typically vary, leading to changes in the α-diversity of soil microorganisms. However, contrary to our initial hypothesis, there were no significant differences in soil bacterial α-diversity among the three different restoration modes (Figure S1). This may be due to the fact that bacteria generally have broader metabolic capabilities and nutritional requirements than fungi, allowing them to utilize a diverse range of organic and inorganic substances [39]. For example, studies have shown that bacteria are generally more efficient than fungi at utilizing simple organic compounds [40]. Bacteria can rapidly utilize simple substrates, such as low molecular weight organics, while fungi have an advantage in utilizing complex compounds [41]. Thus, the increased organic matter and nutrients in the soil can be effectively utilized by bacteria under different restoration modes, thereby promoting their growth and reproduction. As a result, bacterial diversity reached relatively high levels under both restoration regimes. Consistent with our hypothesis, both the Shannon and Simpson indices of soil fungi in the NR were significantly higher than those in the CK and AR (Figure S2). Given the greater abundance of understory plants, such as shrubs, herbs, and mosses, observed in the naturally restored birch forests, we propose that the higher plant species diversity provides a richer source of nutrients and a broader range of ecological niches for soil fungi. This, in turn, promotes the diversity and uniformity of fungal species [42]. In addition, there may be differences in succession times between artificial and natural restoration [43]. As succession progresses, the birch forests under artificial restoration will gradually develop into mature birch forests, eventually becoming the climax community [44]. As we found a small number of Larix gmelinii in the naturally restored birch forests, we speculate that although these forests had also succeeded to the stage of mature birch forests, they continue to succeed to a more advanced mixed birch–larix forest. Previous study confirmed that fungal communities were highly sensitive to their association with forest disturbance and plant diversity [45].

Principal coordinates analysis (PCoA) based on the Bray–Curtis distances revealed significant changes in the structure of soil bacterial and fungal communities among different restoration modes (Figure 1). We postulate that above-ground vegetation composition and environmental conditions were the main driving factors responsible for changes in soil microbial community structure (β-diversity) [46]. We hypothesize that soil microbial communities possess a degree of robustness and resistance to changes in above-ground vegetation composition and environmental conditions associated with artificial restoration have altered the changes in vegetation diversity, lighting, temperature and humidity, resulting in niche differentiation among soil microorganisms and shifts in their life history strategies [47,48]. Soil in artificially restored forests is often subjected to anthropogenic disturbances, such as land clearing, soil tilling, etc., and these activities may have destroyed the original soil structure and microbial communities. As a result, soil organic matter content in artificially restored forests may be low, and nutrient cycling may be less complete [49,50]. In addition, root secretions from artificially restored forests may be more difficult to be effectively utilized by soil microorganisms due to changes in the soil environment, resulting in some of the secretions accumulating in the soil and inhibiting the soil microbial community [51].

### 4.2. Relationship Between Microbiome and Soil Properties

Our study showed that the abundance of the bacterial genus *Rhodoplanes* rose significantly from CK to both NR and AR, along with a marked decline in *Pajaroellobacter* abundance (Figure 3A). Similarly, the fungal genera showed a similar pattern, with significant reductions in the abundance of *Podila* and *Russula* (Figure 3B). The genus *Rhodoplanes* is recognized as a plant growth-promoting rhizobacterium (PGPR) that can efficiently utilize various forms of carbon compounds, facilitating soil carbon and nitrogen cycling [52]. This symbiotic relationship aids in plant growth and health while also providing *Rhodoplanes* with abundant nutrient sources and habitats [53]. Artificial restoration typically involves the planting and management of specific plants whose root exudates may provide suitable growth conditions and nutrient sources for the *Rhodoplanes* genus, explaining why their abundance is highest in the AR. Studies have shown that the genus *Pajaroellobacter* was associated with plant health [54], with the highest abundance observed in the CK, highlighting the importance of normal growth of *Betula platyphylla* forests under natural conditions. However, *Pajaroellobacter* had the second highest abundance in the NR and the lowest in the AR, providing further evidence that the damage to nature caused by human activities cannot be restored by any restoration measures. *Podila minutissima* is a saprotrophic parasitic fungus capable of decomposing dead fungal tissue [55]. This suggests that other species within the genus *Podila* may also possess, to some extent, the characteristics of saprotrophic or parasitic fungi. Due to their distribution in the soil and their mode of life, the genus *Podila* plays an important role in soil ecosystems, particularly in organic matter decomposition and nutrient cycling [56]. The SOC and DOC levels in CK were markedly higher than in other restoration modes, highlighting the *Podila* genus’ key role in carbon and nutrient cycling. Additionally, the *Russula* genus is crucial for forest organic matter decomposition, promoting nutrient cycling and sustaining soil fertility [57,58]. As a naturally mature *B. platyphylla* forest, the CK showed stronger symbiotic relationships with both *Podila* and *Russula* compared to the NR and AR. This again emphasizes the importance of protecting forest resources and reducing forest degradation caused by human activities.

The relative abundance of the top ten bacterial genera does not change significantly (Figure S4A). Among the top ten fungal genera in terms of abundance (Figure S4B), both NR and AR have their unique fungal genera, such as *Piloderma* (a unique genus in the NR), which is an important ectomycorrhizal fungus that forms ectomycorrhizal symbioses with various tree species [59], playing a vital role in promoting plant growth and enabling nutrient uptake [60]. For example, the genus *Piloderma* can obtain nitrogen from organic nitrogen sources such as proteins and provide it to host plants such as *Scots pine* [59]. In this study, we think that the concentrations of TN and AN were higher in the natural restoration (NR) mode compared to the manual restoration mode and this may be the reason why *Piloderma* was a unique genus in NR. Although the genus *Inocybe* was present in small amounts in the NR, its abundance was much higher in the AR compared to that of NR. Many species within the *Inocybe* genus form ectomycorrhizal symbioses with *Betula platyphylla* [61,62]. Ectomycorrhizal fungi are capable of absorbing water and minerals (such as phosphorus and potassium) from the soil [63]. They can also secrete enzymes and organic acids [64] that break down organic matter and insoluble minerals in the soil, converting them into forms that can be directly absorbed by plants [65]. These fungi then transport these nutrients to the plant roots, thereby promoting plant growth [66]. This explains why the levels of AP and AK are higher in the AR than in the NR, and why there is a positive correlation between AP and AK in the AR. Additionally, *Inocybe* genus produces large amounts of organic acids, such as citric acid, malic acid, and oxalic acid, which explains why the AR group had the lowest pH. Therefore, differences in the processes of natural and manual restoration alter the composition of soil microorganisms, which in turn leads to changes in soil physicochemical properties.

There was a strong correlation between soil microbial communities and environmental factors (Figure 5 and Figure 6), with Mucilaginibacter showing a significant positive correlation with SOC, DOC and TN. This might be attributed to Mucilaginibacter’s essential function in breaking down soil organic matter and driving nutrient cycling [30]. Studies have shown that bacteria belonging to the genus Mucilaginibacter have the ability to degrade a variety of complex organic substances. These bacteria facilitate the degradation of organic matter by secreting various enzymes (such as cellulases and pectinases) to break down complex compounds such as xylan and pectin into simpler substances [67]. These organic compounds are important components of soil organic matter, and their degradation helps release nutrients into the soil. In addition, research by Wang and Kuzyakov has shown that *Mucilaginibacter* sp. K promotes plant growth and development by activating signaling pathways of plant hormones such as auxin and gibberellin [41]. These hormones also play a key role in nitrogen metabolism and utilization in plants, indirectly influencing nitrogen use efficiency. The bacterial genus *Geobacter* and the fungal genus *Pseudosperma* both showed positive correlations with AP and AK. *Geobacter* facilitates the mineralization process of phosphorus through its metabolic activities, thereby influencing the distribution and utilization of phosphorus in the environment. For example, in wastewater, the *Geobacter* genus participates in a bio-induced vivianite (a phosphate-iron mineral) recovery process to utilize phosphorus [68]. During this process, the growth of *Geobacter* biomass was associated with phosphorus utilization in the mineralization of phosphorus ores, indicating its important role in phosphorus recovery and cycling [69]. The positive correlation with AK may be attributed to the indirect promotion of potassium release and uptake by plants through the improvement of soil structure and facilitation of organic matter decomposition. *Pseudosperma*, similar to the fungal genus *Inocybe*, typically assists plants in acquiring phosphorus and potassium, especially insoluble phosphorus, from the soil through its ectomycorrhizal fungi [70]. These fungi use their extensive hyphal networks to absorb phosphorus from the soil and convert it into forms that can be used by plants [71].

### 4.3. Assembly Processes (Deterministic vs. Stochastic)

The neutral community model (NCM) is primarily used to quantify the stochastic and deterministic processes in community assembly [32]. Our observations suggest that both soil bacterial and fungal communities are driven by deterministic processes (Figure 8). Deterministic processes involve interspecies interactions like competition, antagonism and exclusion, as well as environmental selection based on habitat conditions. Once initial conditions are established, deterministic processes exhibit subsequent evolutionary trajectories that follow clear and fixed rules, allowing accurate predictions [72]. This is not consistent with our initial hypothesis. However, in all treatment groups, the assembly of bacterial communities from AR to CK gradually approached stochastic processes. In mature birch forests, soil bacterial communities have undergone a long succession and have gradually evolved into a stable community structure. This stability suggests that the community is no longer solely dependent on random dispersal and drift processes, but is increasingly controlled by environmental selection and biological interactions [73]. Our results are consistent with other studies [21,74,75]. For example, in the rhizosphere of birch trees, bacterial communities’ structures are significantly influenced by TN in the soil [74]. In birch rhizosphere soils, the relative abundances of the primary bacterial phyla Proteobacteria, Acidobacteria and Actinobacteria are related to soil TN [76]. These bacteria may gain competitive advantages in specific soil environments and, thus, dominate the community. In contrast to mature birch forests, natural and artificially restored birch forests show legacy effects of past logging and human activities on the current composition of soil bacterial communities. These historical factors influence the stability and resilience of the communities, enabling them to withstand, to some extent, disturbances caused by stochastic processes [77]. For example, other studies conducted have shown significant effects of logging disturbances on the structure and function of temperate forests. Logging changes soil organic carbon levels and soil nutrients such as available nitrogen, phosphorus and potassium [78]. These changes affect the composition and function of soil bacterial communities because logging alters the physicochemical properties of the soil, thereby exerting long-term effects on the stability and resilience of bacterial communities. Additionally, soil cultivation and the planting of a single species in artificial restoration projects tend to homogenize microhabitats, thereby inhibiting random diffusion [5]. In contrast, the assembly of soil fungal communities is strongly influenced by deterministic processes, with the highest degree of community dispersal observed in the NR treatment. This pattern differs from some previous studies [79,80,81]. However, our results are consistent with other studies that indicate that human activities can induce niche competition among soil fungi. Fungi, as K-strategists, have longer generation cycles and lower reproductive rates, allowing them to accumulate adaptive traits over time in stable environments and, thus, gain a competitive advantage [82,83,84]. However, when faced with environmental logging pressures, fungi may be able to use available resources effectively (Figure 7), but may require considerable time to adapt to new environmental conditions in the event of drastic changes. These stressors may trigger environmental filtering and ecological selection, making soil fungal communities more susceptible to deterministic processes. Although both recovery methods reinforce the certainty process, the approaches are different. *Betula platyphylla* Suk. planted in artificial restoration sites preferentially associate with host-specific ectomycorrhizal fungi (e.g., *Inocybe*), which utilize altered nutrient pools (low pH, high AP, and AK) through close symbiotic relationships with plants, thereby achieving deterministic dominance [72]. In contrast, the higher plant diversity in natural restoration creates heterogeneous organic substrates (e.g., diverse litter chemical composition), which promote the deterministic assembly of fungal communities through resource differentiation, including genera such as saprophytic fungi (*Podila*) and mycorrhizal fungi (*Russula*) [82,83]. The high Nm values achieved through natural restoration (96,816 for bacteria and 12,304 for fungi) accelerated community reorganization under natural succession. In contrast, the limited dispersal capacity achieved through artificial restoration prolonged the period of deterministic dominance. This suggests that restoration methods alter current selection pressures and regulate the assembly trajectory of communities by influencing microbial dispersal and residual effects over time.

## 5. Conclusions

Inconsistent with our first hypothesis, our results showed that soil bacterial α-diversity did not change significantly, but soil fungal α-diversity altered significantly among natural restoration. Moreover, inconsistent with the α-diversity of soil microbial (bacterial and fungal) communities, the soil microbial β-diversity of soil bacterial and fungal communities both changed significantly among restoration modes. Our results indicate that the abundance of the bacterial genus *Rhodoplanes* significantly increases under artificial restoration, while the abundance of the bacterial genus *Pajaroellobacter* significantly increases under natural restoration. Conversely, the fungal genera Podila and *Russula* showed a significant decrease in abundance under artificial restoration. These genera are essential in determining the functional traits of soil microbial communities. Moreover, this study made a significant discovery that certain microorganisms, including those from the genera *Geobacter*, *Pseudosperma* and *Inocybe*, play a notable role in helping plants to acquire phosphorus and potassium from the soil through their ectomycorrhizal fungi and convert them into forms that can be used by plants, which explains the positive correlation between AP and AK with artificial restoration treatment groups. In particular, the assembly of soil bacterial and fungal communities under different restoration treatments is driven by deterministic processes, indicating a substantial influence of human activities on microbial distribution and abundance. Based on these findings, we propose the following management recommendations for forest ecosystems: It is imperative to conserve forest resources and minimize disturbance from human activities in forests, as we found that neither natural restoration nor artificial restoration could completely restore the Betula forests to their pre-disturbance state. We can only strive to select restoration methods with minimal impact on nature and proven effectiveness in restoring forest ecosystems as close as possible to their optimal condition. This study builds on existing literature to present new conceptual advances regarding microbial community changes under forest restoration. Not only did we compare the effects of natural and artificial restoration on soil microbial community structure, but we also examined the mechanisms of microbial community assembly under different restoration modes. This revealed the different roles of deterministic and stochastic processes in shaping bacterial and fungal communities. Furthermore, this study emphasizes the unique functional contributions of specific microbial taxa to nutrient cycling and soil health, offering valuable insights for the sustainable management and restoration of forests.

## Figures and Tables

**Table 1:** Chemical attributes of soil under different treatments.

Sample	SOC (%)	DOC (g/kg)	TN (g/kg)	AN (mg/kg)	AP (mg/kg)	AK (mg/kg)	pH
CK	11.05 ± 0.08 a	87.79 ± 1.57 a	5.15 ± 0.2 a	399.5 ± 11.17 a	10.52 ± 0.54 b	12.59 ± 0.46 b	5.74 ± 0.06 b
NR	2.28 ± 0.12 b	27.99 ± 1.46 b	1.87 ± 0.1 b	186.61 ± 2.66 b	9.31 ± 0.18 b	11.07 ± 0.25 c	6.09 ± 0.05 a
AR	2.02 ± 0.1 c	24.7 ± 0.85 c	1.39 ± 0.05 c	124.76 ± 8.92 c	37.14 ± 1.24 a	17.7 ± 0.23 a	5.54 ± 0.03 c

Note: CK: Naturally mature forests. NR: Naturally restored forests. AR: Artificially restored forests. For each treatment, three replications were executed. The data are presented as the mean with the standard deviation, and lowercase letters denote significant differences (*p* < 0.05). Total nitrogen (TN), alkaline hydrolysable nitrogen (AN), dissolved organic carbon (DOC), soil organic carbon (SOC), available phosphorus (AP) and quick-acting potassium (AK).

**Table 2:** Bacterial and fungal communities’ α-diversity in different treatments.

Microbial Community	Sample	Shannon Index	Simpson Index	Ace Index	Chao1 Index
Bacteria	CK	5.866 ± 0.066 a	0.983 ± 0.003 a	4301.782 ± 96.052 a	4213.372 ± 98.843 a
	NR	6 ± 0.137 a	0.987 ± 0.003 a	4932.454 ± 912.191 a	4801.497 ± 1013.265 a
	AR	5.756 ± 0.135 a	0.977 ± 0.007 a	4285.922 ± 193.553 a	4164.067 ± 239.993 a
Fungi	CK	2.938 ± 0.172 b	0.81 ± 0.04 b	710.818 ± 30.715 a	721.697 ± 37.061 a
	NR	3.984 ± 0.259 a	0.957 ± 0.01 a	792.697 ± 96.283 a	808.458 ± 95.75 a
	AR	3.084 ± 0.235 b	0.843 ± 0.04 b	684.046 ± 32.371 a	704.565 ± 47.596 a

Note: CK: Naturally mature forests. NR: Naturally restored forests. AR: Artificially restored forests. Each treatment was replicated three times as PCR technical replicates to ensure experimental reliability. Data are presented as the mean ± standard deviation, with lowercase letters used to denote significant differences between groups. (*p* < 0.05).

## Data Availability

Data are contained within the article.

## Supplementary Materials

The following supporting information can be downloaded at: https://www.mdpi.com/article/10.3390/microorganisms13061339/s1, Figure S1: The diversity indices of soil bacterial communities under different restoration methods.; Figure S2: The diversity indices of soil fungal communities under different restoration approaches.; Figure S3: Venn diagrams and bar charts of soil bacterial (A) and fungal (B) operational taxonomic units (OTUs) under different forest restoration strategies.; Figure S4: The Circos diagram of bacterial genera (A) and fungal genera (B) under different restoration modes.; Figure S5: The top ten abundant soil bacterial phyla (A) and soil fungal phyla (B) under varying treatments are illustrated by bar charts.; Figure S6: Changes in soil physicochemical properties under different forest restoration strategies.; Figure S7: The variation of soil pH.; Figure S8: Heatmap of soil bacterial functional gene relative abundance under different forest restoration strategies.; Figure S9: Heatmap of soil fungal community functional types relative abundance under different forest restoration strategies.
