# Supplementary material for: Changes in Soil Microbial Community Structure and Assembly Process Under Different Forest Restoration Strategies in Cold Temperate Forests of Northeastern China
Source: Microorganisms. 2025 Jun 9;13(6):1339. doi: 10.3390/microorganisms13061339 (PMC12195559; doi:10.3390/microorganisms13061339)
Supplement: Supplementary file 1 [file microorganisms-13-01339-s001.zip › microorganisms-3610763-supplementary.pdf]

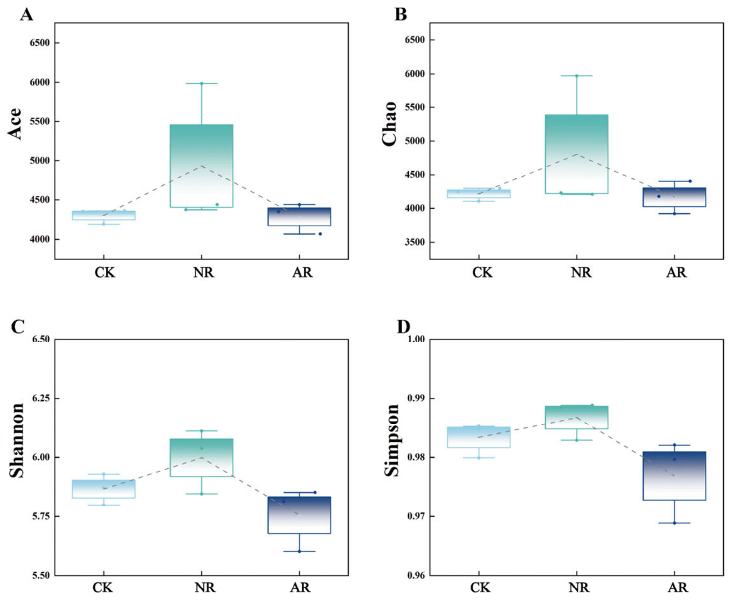

Figure S1. The diversity indices ACE (A), Chao1 (B), Shannon (C), and Simpson (D) of soil bacterial communities under different restoration methods. CK: Naturally mature forests. NR: Naturally restored forests. AR: Artificially restored forests.

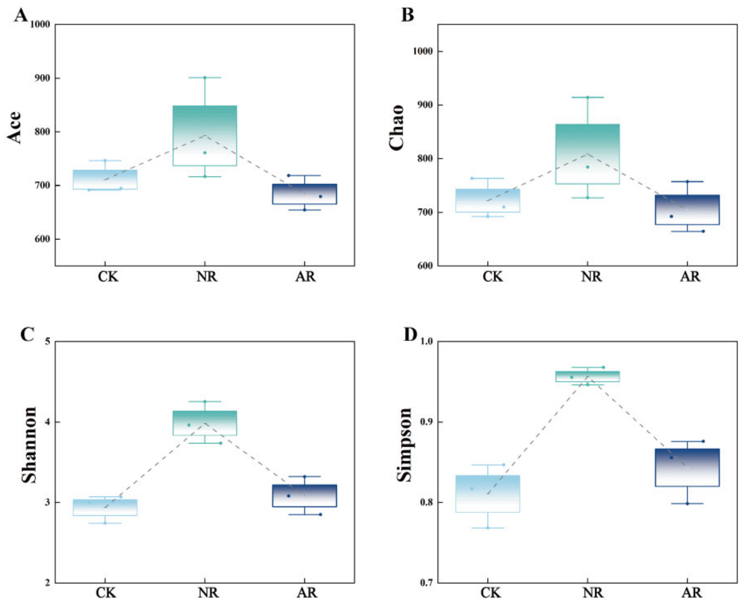

Figure S2. The diversity indices ACE (A), Chao1 (B), Shannon (C), and Simpson (D) of soil fungal communities under different restoration approaches. CK: Naturally mature forests. NR: Naturally restored forests. AR: Artificially restored forests.

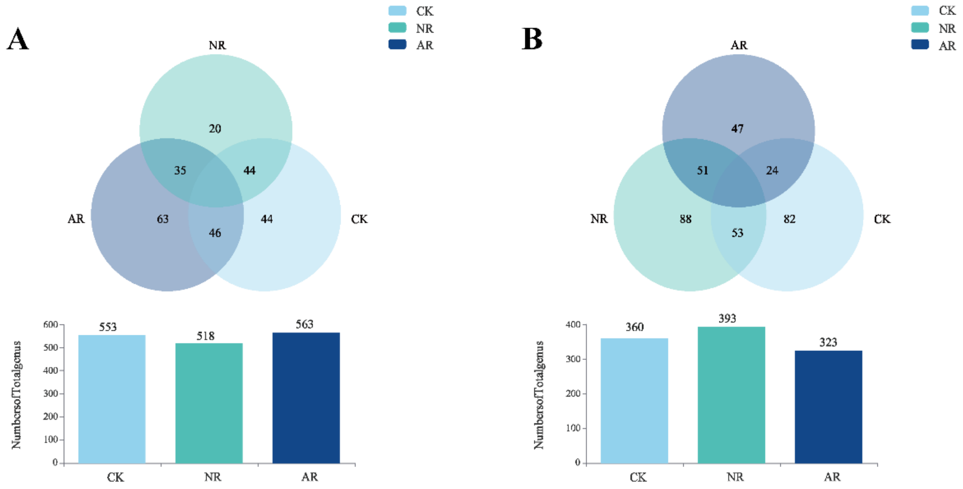

Figure S3. Venn diagrams and bar charts of soil bacterial (A) and fungal (B) operational taxonomic units (OTUs) under different forest restoration strategies. When the number of groups (or samples) is ≤5, the graph will display a Venn diagram, where different colors represent different groups (or samples). The numbers in the overlapping sections indicate the number of species shared among multiple groups (or samples), while the numbers in the non-overlapping sections represent the species unique to the corresponding group (or sample). When the number of groups (or samples) is between 6 and 10, the graph will display an UpSet plot, where the left coordinate chart represents the total number of species, and the middle bar chart indicates the number of unique species. When the number of groups (or samples) exceeds 10, the graph will display a petal diagram, where the petals contain the number of species unique to the corresponding group (or sample), the center represents the number of species shared by all groups (or samples), and the bar chart below shows the total number of species for each sample. CK: Naturally mature forests. NR: Naturally restored forests. AR: Artificially restored forests.

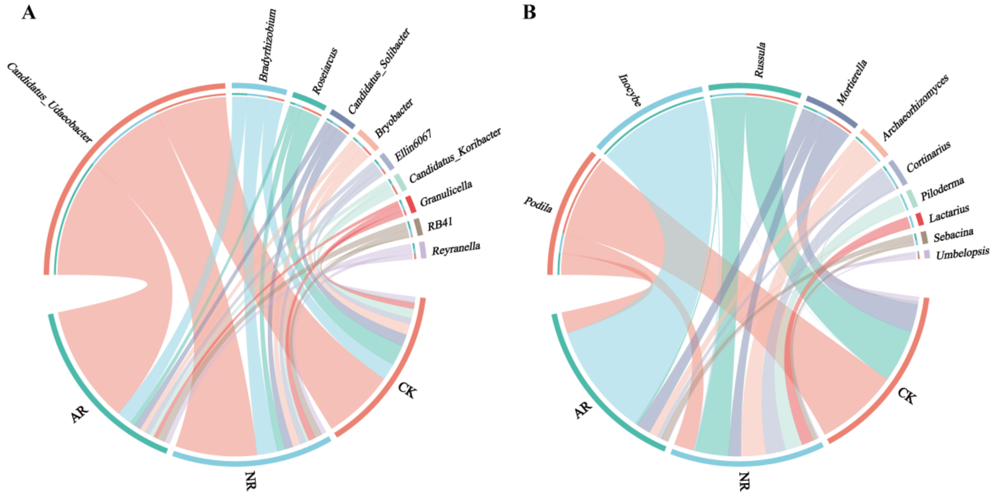

Figure S4. The Circos diagram intuitively shows the similarities and differences in the composition of bacterial genera (A) and fungal genera (B) under different restoration modes. In the figure, the outer circle marks different microbial genera, and the inner circle indicates the coexistence relationship of

different microbial genera under different recovery modes through connecting lines. CK: Naturally mature forests. NR: Naturally restored forests. AR: Artificially restored forests.

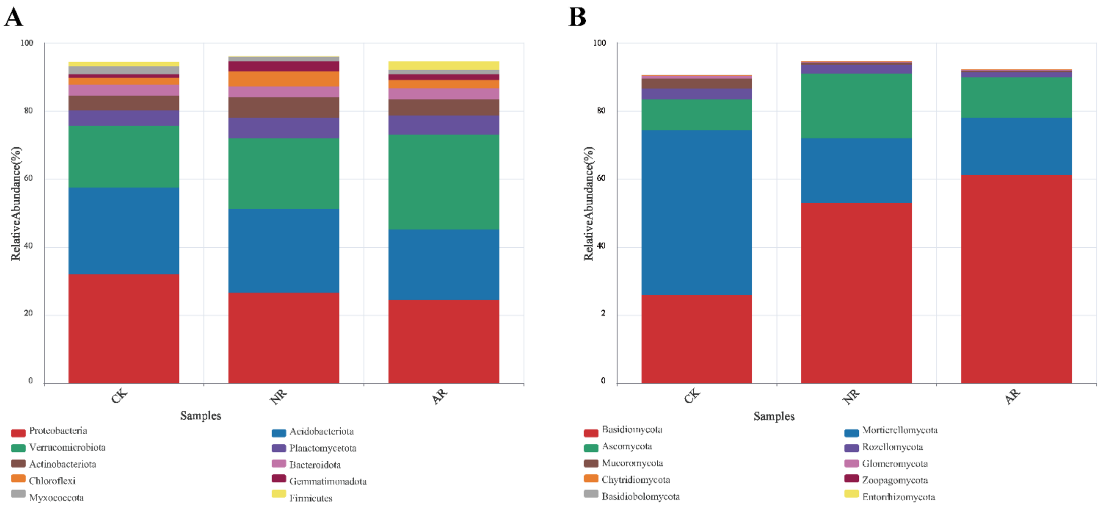

Figure S5. The top ten abundant soil bacterial phyla (A) and soil fungal phyla (B) under varying treatments are illustrated by bar charts. The horizontal axis represents the sample numbers, while the vertical axis indicates the relative abundance proportion of species. The colors correspond to the names of phyla at this taxonomic level, and the width of different color blocks represents the relative abundance proportion of different species. CK: Naturally mature forests. NR: Naturally restored forests. AR: Artificially restored forests.

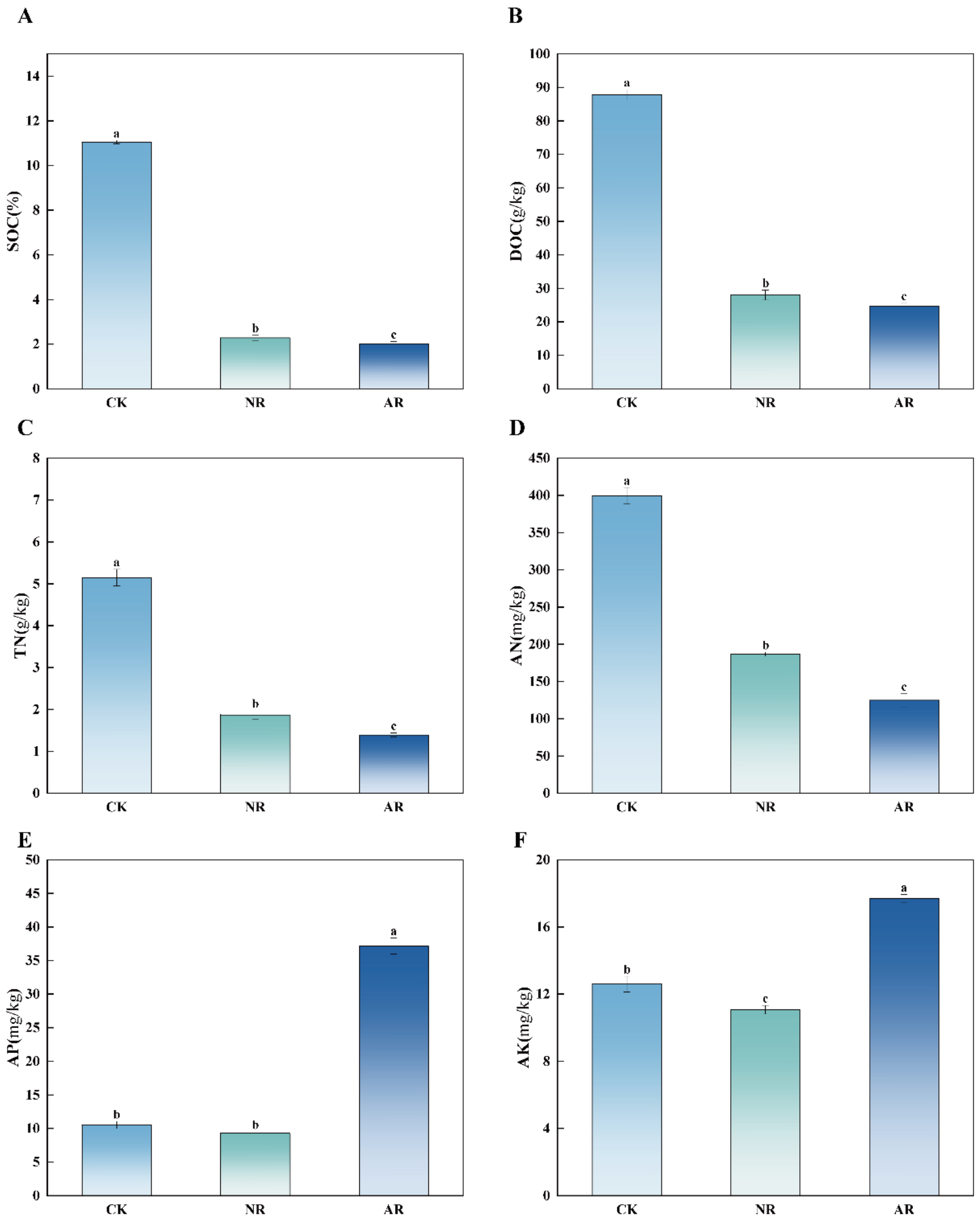

Figure S6. Changes in soil physicochemical properties under different forest restoration strategies.This figure shows the concentrations of soil organic carbon (SOC), dissolved organic carbon (DOC), total nitrogen (TN), alkaline dissolved nitrogen (AN), available phosphorus (AP), and quick-acting potassium (AK) in natural mature forests (CK), naturally restored forests (NR), and artificially restored forests (AR). Data were obtained by standard soil chemical analysis methods. Each bar represents the mean concentration (± standard deviation) of each parameter based on three replicate samples. y-axis represents the concentration of each soil attribute in the specific units indicated next to each parameter. Significant differences between treatment groups

are indicated by lower case letters (P < 0.05).

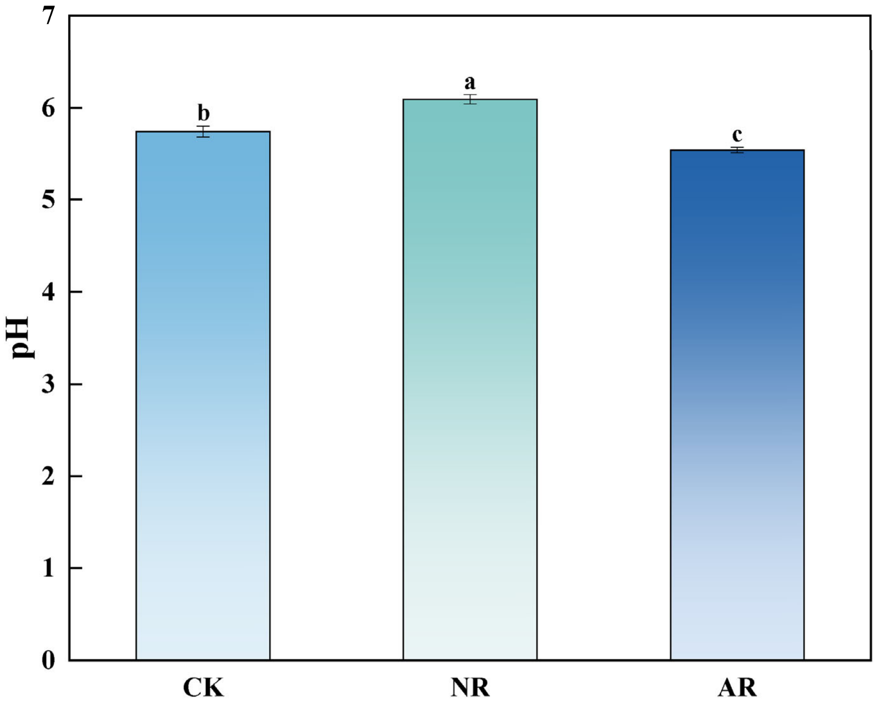

Figure S7. This figure shows the variation of soil pH in natural mature forest (CK), naturally restored forest (NR) and artificially restored forest (AR). Each bar represents the mean pH (± standard deviation) of three replicate samples. Significant differences between treatment groups are indicated by lower case letters ($P < 0.05$).

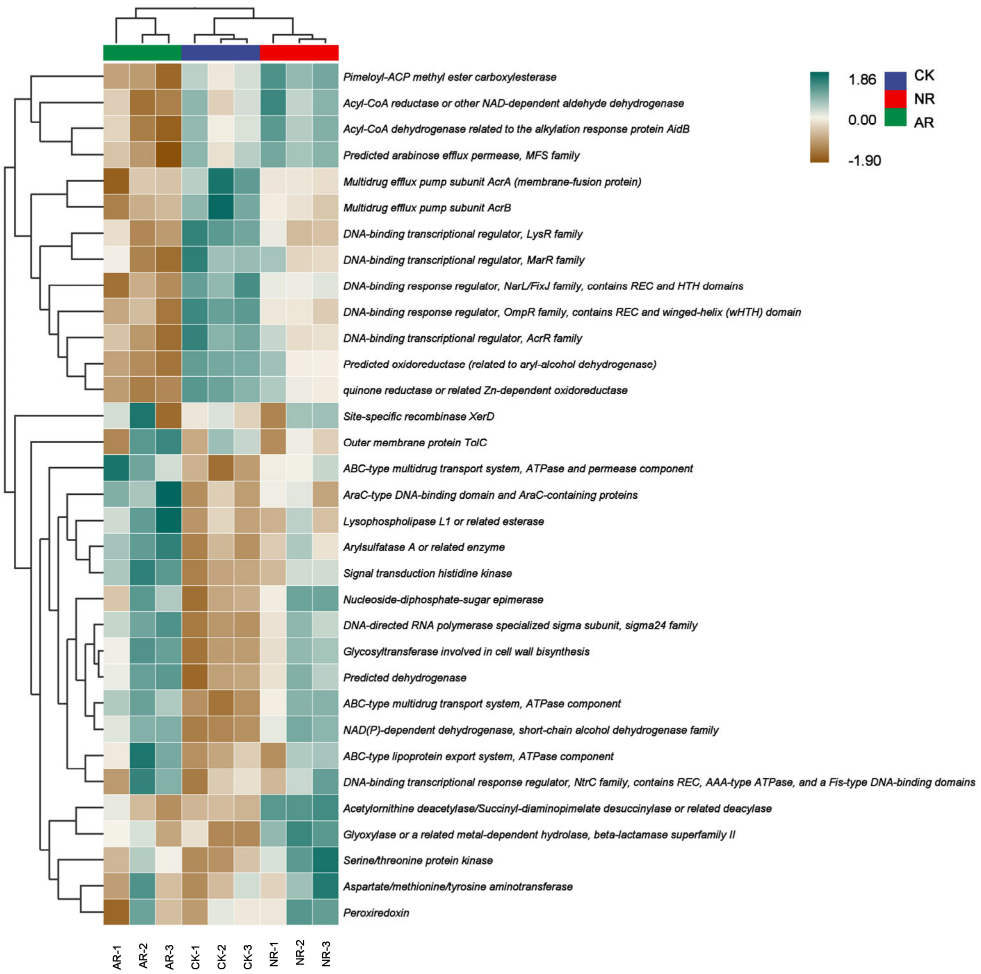

Figure S8. Heatmap of soil bacterial functional gene relative abundance under different forest restoration strategies. This heatmap illustrates the relative abundance of soil bacterial functional genes across naturally mature forests (CK), naturally restored forests (NR), and artificially restored forests (AR). The color gradient ranges from brown (low abundance) to green (high abundance), with CK, NR, and AR represented by blue, red, and green, respectively.

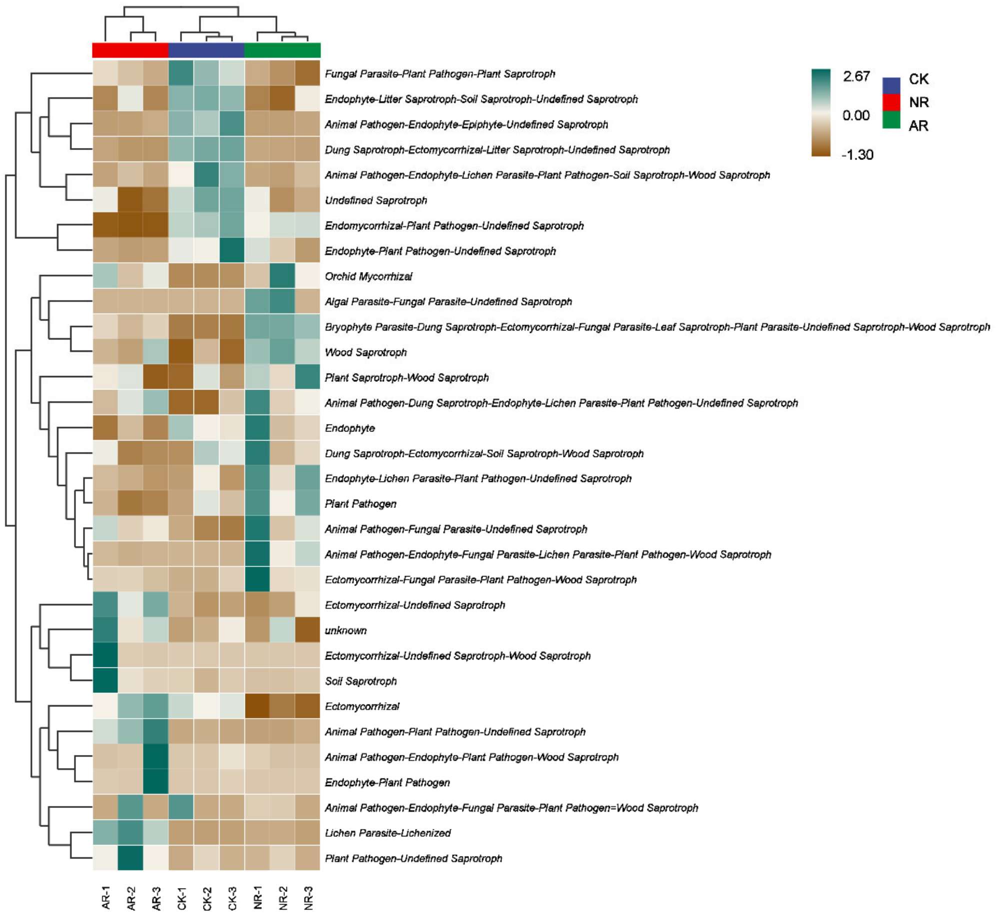

Figure S9. Heatmap of soil fungal community functional types relative abundance under different forest restoration strategies. This heatmap illustrates the relative abundance of functional types of soil fungal communities across naturally mature forests (CK), naturally restored forests (NR), and artificially restored forests (AR). The color gradient ranges from brown (low abundance) to green (high abundance), with CK, NR, and AR represented by blue, red, and green, respectively.
